# American Neurological Association

**Published:** 1892-07

**Authors:** 


### Society Meetings.

The eighteenth annual meeting of the American Neurological Association was held at the New York Academy of Medicine, June 22, 23, and 24, 1892. Among the papers read were those by Dr. J. W. Putnam, of Buffalo, on Sleep Movements of Epilepsy, and by Dr. Wm. C. Krauss, of Buffalo, on The Use of Exalgine in Painful Nervous Affections, and another on Two Cases of Severe Pressure Neuritis.

